# DiffraGAN: a conditional generative adversarial network for phasing single molecule diffraction data to atomic resolution

**DOI:** 10.3389/fmolb.2024.1386963

**Published:** 2024-05-22

**Authors:** S. Matinyan, P. Filipcik, E. van Genderen, J. P. Abrahams

**Affiliations:** ^1^ Biozentrum, Basel University, Basel, Switzerland; ^2^ Paul Scherrer Institute, Villigen, Switzerland

**Keywords:** diffraction, cryo-EM, deep learning, generative adversarial network, simED

## Abstract

**Introduction:**

Proteins that adopt multiple conformations pose significant challenges in structural biology research and pharmaceutical development, as structure determination via single particle cryo-electron microscopy (cryo-EM) is often impeded by data heterogeneity. In this context, the enhanced signal-to-noise ratio of single molecule cryo-electron diffraction (simED) offers a promising alternative. However, a significant challenge in diffraction methods is the loss of phase information, which is crucial for accurate structure determination.

**Methods:**

Here, we present DiffraGAN, a conditional generative adversarial network (cGAN) that estimates the missing phases at high resolution from a combination of single particle high-resolution diffraction data and low-resolution image data.

**Results:**

For simulated datasets, DiffraGAN allows effectively determining protein structures at atomic resolution from diffraction patterns and noisy low-resolution images.

**Discussion:**

Our findings suggest that combining single particle cryo-electron diffraction with advanced generative modeling, as in DiffraGAN, could revolutionize the way protein structures are determined, offering an alternative and complementary approach to existing methods.

## 1 Introduction

Single particle cryo-electron microcopy (cryo-EM) allows resolving the structure of macromolecular complexes with near atomic resolution (Zhang et al., 2008; Nakane et al., 2020; Yip et al., 2020). However, visualizing such biological complexes faces certain limitations. These include the very poor contrast of proteins, the need for low electron dose conditions to prevent radiation damage to the proteins, and the thickness of the ice encasing the specimens (Bepler et al., 2020). The expected signal-to-noise ratio (SNR) of a cryo-EM micrograph is estimated to be only as high as 0.1 (Baxter et al., 2009). While the SNR of an image can be improved by increasing the incident dose, this would destroy the macromolecule long before a sufficient number of scattering events is detected for a high-resolution structural analysis (Miao et al., 1999). These issues severely complicate structural analysis of small proteins, or of dynamic protein complexes that are present in many different conformations. In these cases, the signal becomes increasingly difficult to distinguish from noise.

We are analyzing far-field electron scattering diffraction data generated by diffracting a 10–45 nm narrow, parallel electron beam on a protein sample. Assuming the beam is not much wider than the size of the protein of interest, this approach is likely to improve the SNR ratio compared with cryo-EM imaging (Matinyan et al., 2023). Reportedly, much higher SNRs are observed when collecting data in this mode (Latychevskaia and Abrahams, 2019). However, measuring the diffracted wave function directly as a diffraction pattern has downsides.

In single particle cryo-EM the diffracted wave function is focused back into the image plane, providing phase information through phase contrast. The contrast observed in such images gives insights into the variations in electron density within the sample, and consequently, its structure (Clabbers and Abrahams, 2018). In the case of diffraction data, the phase information becomes much harder to retrieve, especially when the samples are complex molecules such as proteins. With the methodology outlined above, there is no easy and precise way to obtain the phase information of an electron wave function recorded in the diffraction plane. Here, we explore a computational approach for phase retrieval using neural networks, an approach uniquely suited to analysis of complex, multi-dimensional data. Neural networks of diverse topologies have been employed with great success in many areas of image analysis. Mirroring the architecture of biological neural networks, these computational models consist of interconnected neurons with learnable weights. Through iterative optimization, these networks are trained to minimize a loss function, aligning the model’s predictions with a target domain. Among the various neural network architectures, convolutional neural networks (CNN) are particularly tailored for image data handling. Unlike standard feed-forward neural networks, CNNs incorporate specialized layers, such as convolution and pooling, to process spatial hierarchies in the input data. This design enables the network to recognize spatial patterns in the image, known as receptive fields, by selectively weighting neurons based on the significance of different portions of the input (LeCun et al., 1989).

**FIGURE 1:**
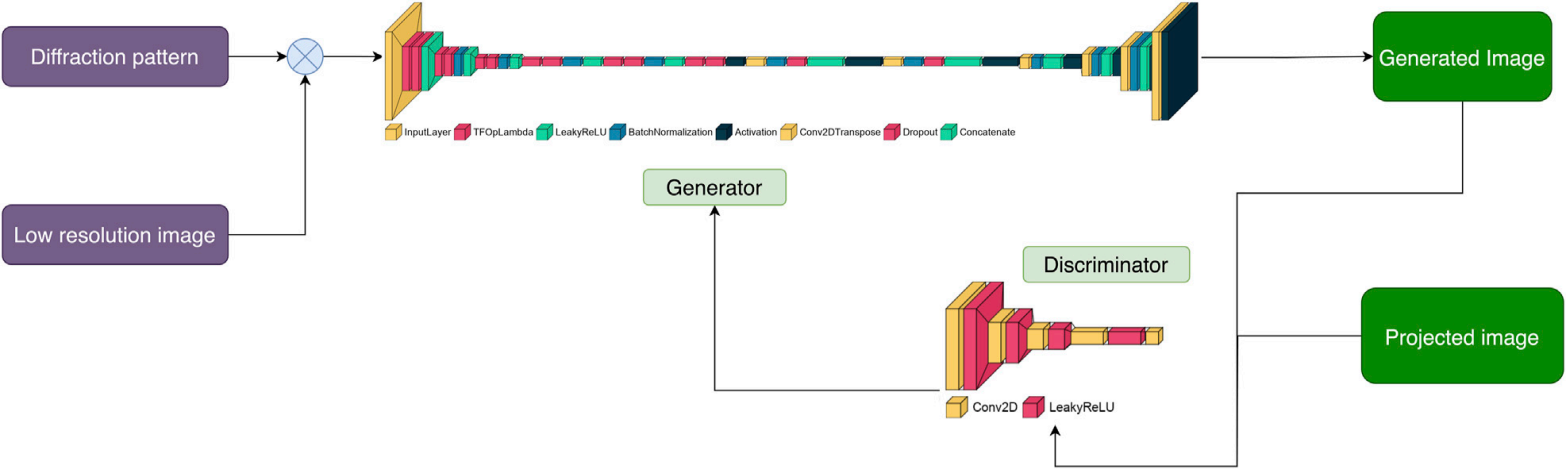
Summary of DiffraGAN training procedure. Models of proteins were rotated to 1078 different angles, with a new pdb file of the protein saved at each angle. A diffraction pattern, high-resolution projection image, and low-resolution image of the protein in each of the poses were generated using multislice calculations from abTEM. These data were then used for DiffraGAN training.

Here, we have used conditional generative adversarial neural networks (GANs), which typically involve a pair of CNNs, with the purpose of generating the phase information that is missing in single molecule diffraction data (simED). We assumed an experimental setup in which diffraction data are collected by orthogonally scanning a sample with a narrow beam, and subsequently recording a low-resolution overview image of the scanned patch. We also assumed it would be feasible to correlate the diffraction patterns to locations in the image and identify which diffraction patterns belong to protein. We have established such an experimental setup, which combines software that is distributed by the hardware manufacturers JEOL, Amsterdam Scientific Instruments (ASI), and CEOS GmbH, and we are continuing to improve our setup. However, our setup is evolving rapidly and has not yet reached a stable state. In its current state, its technical details are beyond the scope of this paper, and by the time of publication would be superseded by improved versions. We trained a conditional GAN with simulated diffraction data of 20 different proteins, each in 1078 orientations, with the corresponding high-resolution projections as desired outcome (Figure 1). Additionally, the network was given simulated defocused, low-resolution, noisy images of the proteins corresponding to each diffraction pattern. The resulting conditional GAN was successfully capable of phasing high-resolution diffraction data using noisy, low-resolution images of test proteins that were not included in the training.

## 2 Materials and methods

Conditional GANs consist of two CNNs known as the generator and the discriminator. In standard GANs (Goodfellow et al., 2014), the generator’s role is to learn how to convert a random noise vector *x* into an output image *y*. Conditional GANs, however, enhance this process by requiring the generator to learn from both a random noise vector and a specific input image. This method allows the generator to understand and replicate the structured aspects of the input, effectively penalizing any inaccuracies in the combined features of the generated output.

The discriminator, which is another CNN, plays a crucial role in evaluating the generator’s outputs. It is trained adversarially to distinguish between genuine images and the “fakes” produced by the generator. The goal of the generator is to create images so convincing that the discriminator cannot tell them apart from real ones. This dynamic competition improves the generator’s ability to produce highly realistic images, enhancing the overall performance of the conditional GAN. The objective of conditional GAN is to minimize the loss function expressed as:

$$L_{cGAN}\left(C,D\right)=E_{x,y}\left[\log D\left(x,y\right)\right]+$$

$$E_{x,z}(\log(\left.\left.1-D\left(x,E\left(x,z\right)\right)\right]\right]$$

where *x* is the observed image, *y* is the target image, and *z* is the random noise vector. C tries to minimize the objective against adversarial D, which tries to maximize it.

### 2.1 The generator

In our generator, all data from the input image end up going through the narrowest part of the network, forming a “U-Net” architecture. Sometimes it can be beneficial for the GANs performance to skip the narrowest part(s) of the generator altogether by allowing straight connections between the early and late layers (Isola et al., 2016). Specifically, we added a skip connection between ι and n-ι layers, where n is the total number of the layers, thus forming a “U-Net” with skip connections. The common justification for allowing such skips, is that they may preserve and propagate larger elements in the input image’s structure.

### 2.2 The discriminator

**FIGURE 2:**
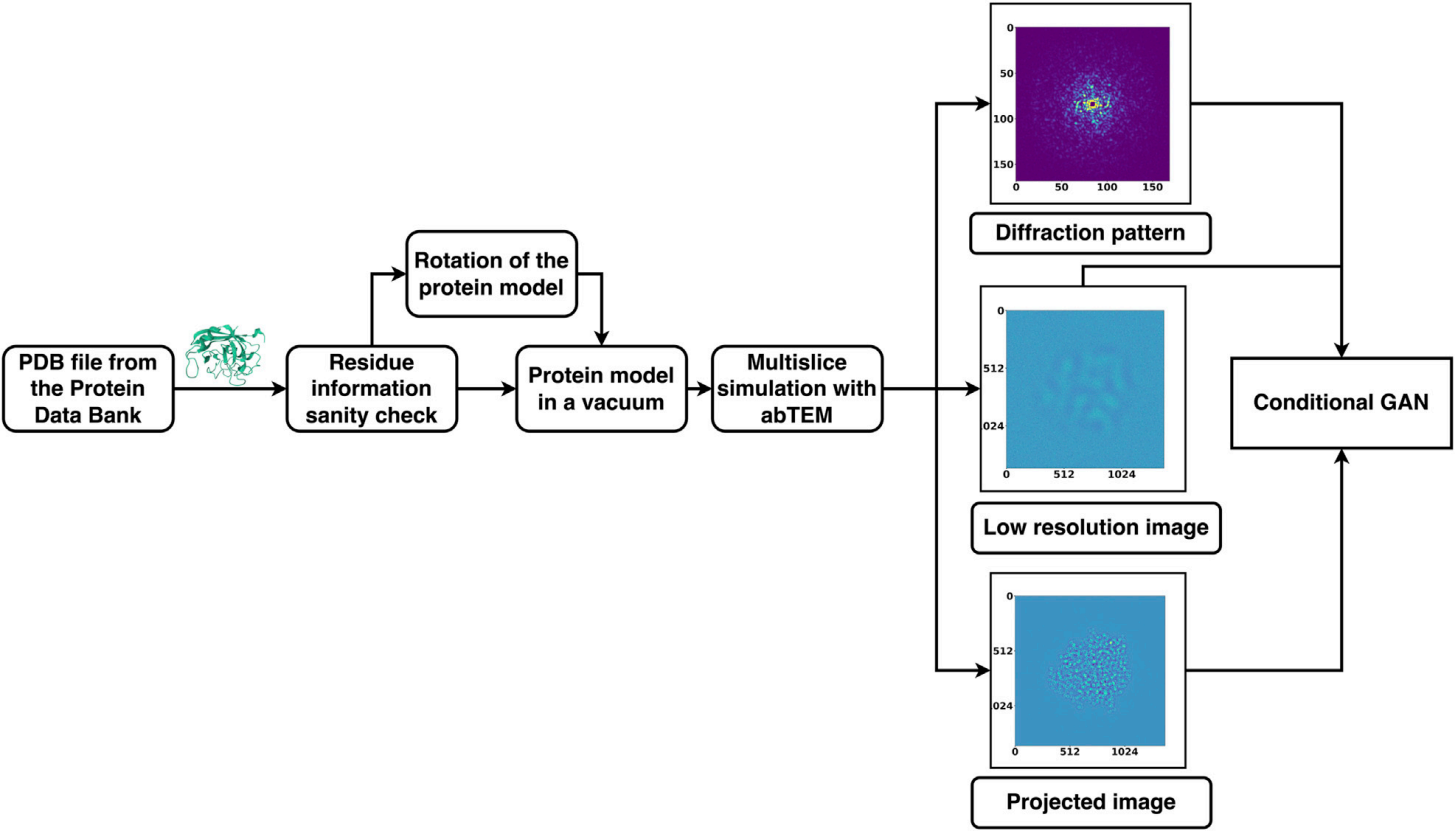
Pairs of diffraction patterns and low-resolution images were given to the generator that was designed to have a U-Net shape. The generator then generates an image conditioned on the input data. Both the generated and non-generated images are given to the discriminator. The discriminator labels each of these images as “real” or generated using the activation map. The more accurate the discriminator is at labelling images, the higher the loss of the generator. The objective of DiffraGAN is to minimize the loss of the generator, i.e., to make the generated images indistinguishable from the high-resolution images.

The discriminator evaluates the generated images and marks them as “real” or generated based on binary cross-entropy (BCE) loss. Our discriminator takes account the low-resolution and generated images, and the high-resolution projections. Inspired by PatchGAN (Isola et al., 2016), where the discriminator penalizes the structure at a scale of predefined patches, our discriminator also makes n number of decisions per image. It divides each image given to it into a specific number of “patches” of pixels, labeling each patch as “real” or generated. The final output of the discriminator is the average of all responses. We also implemented L1 loss to preserve low-resolution details. This term scales the loss of the generator according to the difference between corresponding “real” and generated pixels. The diagrams of the underlying discriminator and generator models are shown in Figure 2.

### 2.3 Datasets and data preparation

Below is a list of the proteins our conditional GAN (DiffraGAN) was trained on (the PDB entry ID for each protein is written in brackets next to it). The pdb files were downloaded from the Protein Data Bank (PDB) and fixed with the python package PDBFixer^1^ (Eastman et al., 2017). Specifically, we replaced nonstandard residues by their standard equivalents, removed all remaining heterogens, added missing hydrogen atoms and deleted water molecules. In cases the proteins were composed of multiple chains, only chain A was used. Each of these proteins was rotated around the center of mass, resulting in 1078 different rotation angle combinations. For each rotation, a new .pdb file was saved. 20 proteins were used for training and 5 proteins for test purposes (Table 1). The test proteins had no sequence similarity (they could not be aligned using the BLOSUM62 substitution matrix to account for evolutionary amino acid substitutions, with minimal gap opening and extension penalties) to the proteins that were used for DiffraGAN training.

**TABLE 1:** The protein.pdb files that were divided into training and test parts. The saved DiffraGAN generator weights have been used to generate images from diffraction patterns and low-resolution images of the test proteins.

PDB entries used	ID	Total structure weight	Resolution
Train dataset			
Apo CopZ from *Bacillus subtilis*	1P8G	7.8 kDa	NMR (Conformer 1)
Cyanobacterial copper metallochaperone, ScAtx1	1SB6	6.69 kDa	NMR (Conformer 1)
C-terminal Domain (537–610) of Human Heat Shock Protein 70	2LMG	8.38 kDa	NMR (Conformer 1)
Ubiquitin	1UBQ	8.58 kDa	1.80 Å
Monoclinic turkey egg lysozyme	135L	14.23 kDa	1.30Å
T4 lysozyme	137L	37.38 kDa	1.85 Å
Profilin I from *Arabidopsis thaliana*	1A0K	14.28 kDa	2.20Å
Peptidylprolyl isomerase, cyclophilin-like domain from *Brugia malayi*	1A33	19.5 kDa	2.15 Å
Ornithine carbamoyltransferase from pyrococcus furiosus	1A1S	35.1 kDa	2.70 Å
Endoglucanase cel5a from *bacillus* agaradherans	1A3H	33.65 kDa	1.57 Å
Tyrosine phosphatase 1b	1A5Y	38.47 kDa	2.15 Å
Human UBC9	1A3S	18.17 kDa	2.80 Å
Cyclophilin from *Brugia malayi*	1A58	19.5 kDA	1.95Å
Gamma s crystallin c-terminal domain	1A7H	20.64 kDa	2.56 Å
Fusarium solani cutinase	1AGY	20.83 kDa	1.15 Å
Ribonuclease A	1AFU	27.42 kDa	2.00 Å
Glutaminase-asparaginase of *acinetobacter* glutaminasificans	1AGX	35.52 kDa	2.90 Å
Top domain of african horse sickness virus vp7	1AHS	40.33 kDa	2.30 Å
Type I fructose 1,6-bisphosphate aldolase	1ALD	39.34 kDa	2.00 Å
Human CD40 ligand	1ALY	15.81 kDa	2.00 Å
Test dataset			
Glutamate dehydrogenase	1AUP	49.21 kDa	2.50Å
Proteinase K	6CL8	28.93 kDa	2.00 Å
Thaumatin	5K7Q	22.23 kDa	2.5 Å
Yeast Sti1 DP1 domain	2LLV	7.94 kDa	NMR (Conformer 1)
Yeast Sti1 DP2 domain	2LLW	7.93 kDa	NMR (Conformer 1)

### 2.4 Multislice simulation

The electron wave function describes the probability of finding an electron at a particular point in space. An electron wave function passing through matter, such as a protein, is diffracted, and analyzing such diffraction patterns can reveal the protein’s structure. The diffraction pattern, the low-resolution and high-resolution projection image pairs of the protein in each of the .pdb files were created by multislice simulation as implemented in the abTEM package (Madsen and Susi, 2021). In abTEM, a complex array on a grid represents the plane wave function of the electron beam. An electron beam interacts with a specimen through the Coulomb potential of its electrons and nuclei. To calculate the electrostatic potential of the sample the independent atom model was used, which neglects any bonding effects and treats the sample as an array of atomic potentials. The wave function is passed slice-by-slice forward along the optical axis of the potential object, yielding an exit wave.

The absolute square of the discrete Fourier transform of the exit wave, yields the intensity distribution in diffraction plane. The high-resolution projection image was calculated as a convolution of the exit wave with a modelled CTF function with a defocus of −50 Å. The low-resolution, defocused images were simulated using a defocus value of −1,000Å and an envelope function with a cutoff at 5 mrad. The diffraction patterns contain information of up to 20 mrad. The simulation and further image processing resulted in real space sampling of 0.27 Å per pixel for high-resolution projection images. The low-resolution images were further degraded by including Poisson noise, which was imposed by altering the irradiation dose per Å^2^ until they were almost indistinguishable from a typical cryo-EM particle-image, however with higher SNR (∼0.8 for the resolution bin of 8 to 5 Å) (Heymann, 2022).

The resulting dataset consists of 25 × 1078 diffraction, low-resolution and high-resolution image triplets, each triplet corresponding to one of the 25 proteins rotated by specific angle. Five of the proteins were used for validation and excluded from the training (Table 1). So far the training has been done with protein molecules that were simulated in vacuum. Details of the training of our DiffraGAN are described as in Figure 2.

To create the results shown below, DiffraGAN was trained using the Adam gradient descent algorithm (Kingma and Ba, 2015), with a learning rate of 0.00002 and with β_1_ parameter of 0.5. The training was performed on the sciCORE high-performance computing (HPC) platform of the University of Basel. Our final model was a 16-pixel patch DiffraGAN and further adjustments of the patch size did not improve the results significantly.

## 3 Results

Since there is no objective loss function to train GANs, the performance of DiffraGAN had to be evaluated using the quality of the generated synthetic images. By considering different aspects of the images, from overall visual appearance to detailed statistical distributions, the evaluation process should provide a comprehensive assessment of the effectiveness of the image generation process. Qualitatively, it is clear when the generator is not working as expected when images do not correspond to the ground truth, and somebody observing two sets of generated images can tell which set matches the target set better “by eye.”

**FIGURE 3:**
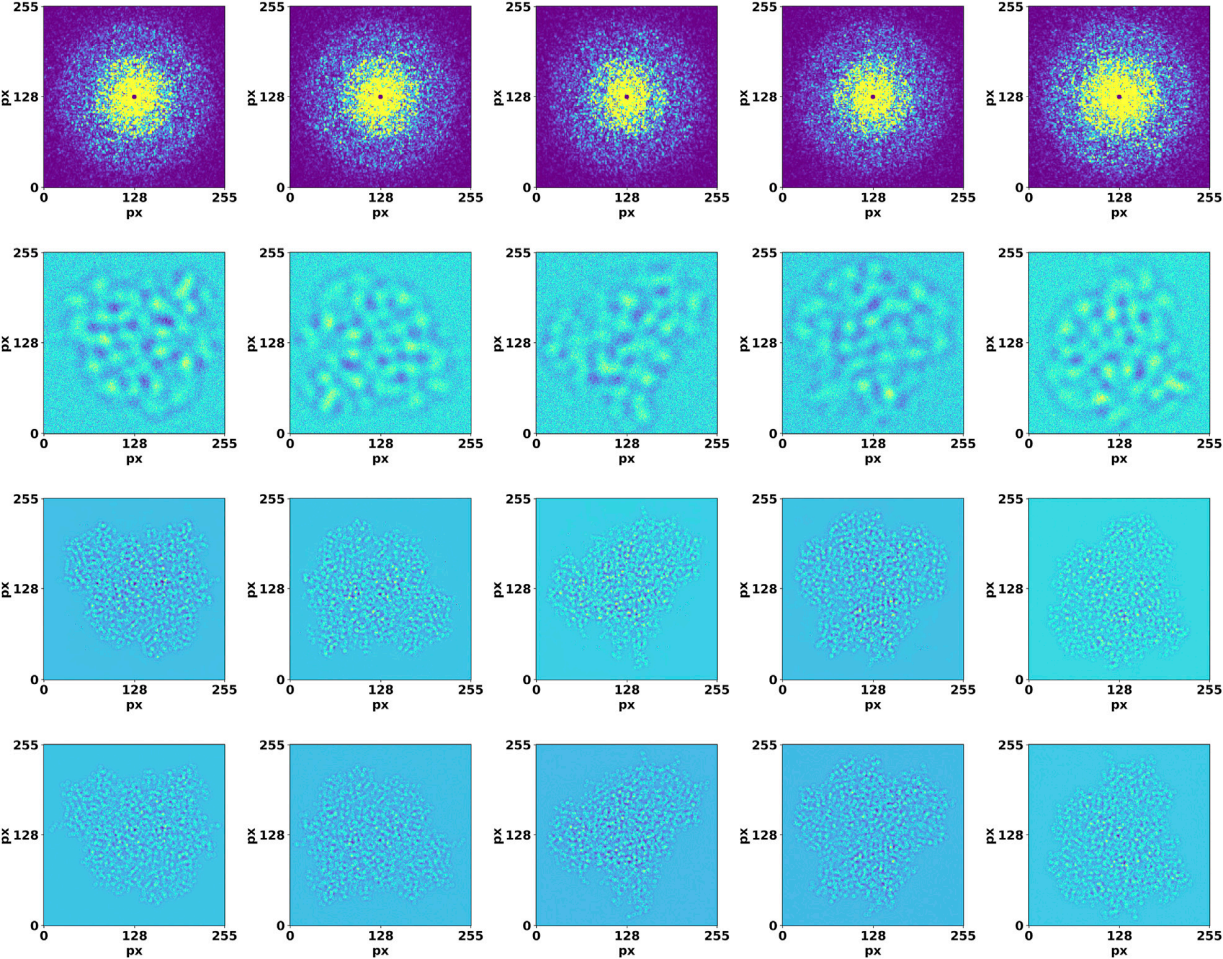
Validation of DiffraGAN using diffraction and image data that were not used in the training. Top row: high-resolution diffraction patterns. Second row: low-resolution, defocused images. Third row: images generated by the generator using these inputs. Bottom row: target images. The axes show pixel number.

**FIGURE 4:**
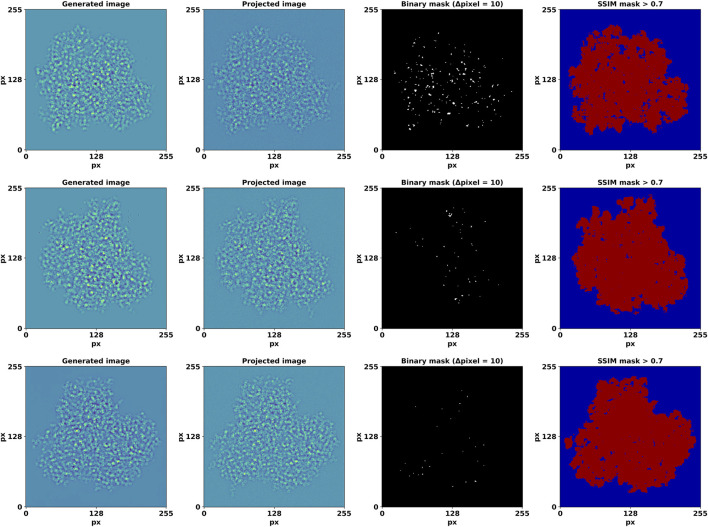
Generated, high-resolution projection image pairs and the detected edges of their differences. The last column depicts regions with an SSIM value > 0.7. The axes show pixel number.

DiffraGAN was trained for 200 epochs using a data set of 20 proteins until the model reached an equilibrium. To evaluate the differences between high-resolution projected and corresponding generated images of the first test protein (PDB ID: 1AUP), a comprehensive comparison was conducted (Details of other test proteins are available in Supplementary Material). Both sets of images were resized to a uniform resolution of 256 × 256 pixels, and then randomly selected pairs were analyzed. The high-resolution projected and generated images were first visually compared to provide an initial assessment of their similarity (Figure 3). To further quantify the differences, a mask was computed using a threshold (Δpixel = 10) on the smoothened absolute differences between corresponding pixels in the generated and projection images. This multi-step process captures the regions where pixel differences are most pronounced, offering a nuanced view of the spatial structure of discrepancies (Figure 4).

We also calculated structural similarity index (SSIM) as more perceptually relevant than other measures (Bakurov et al., 2022). SSIM incorporates perceptual phenomena, and is calculated as:

$$\text{SSIM}\left(x,y\right)=\left(\frac{\left(2\mu_{x}\mu_{y}+C_{1}\right)\left(2\sigma_{xy}+C_{2}\right)}{\left(\mu_{x}^{2}+\mu_{y}^{2}+C_{1}\right)\left(\sigma_{x}^{2}+\sigma_{y}^{2}+C_{2}\right)}\right)$$

Where: $\mu_{x}$ is the average of *x*, $\mu_{y}$ is the average of *y*, $\sigma_{x}^{2}$ is the variance of *x*, $\sigma_{y}^{2}$ is the variance of *y*, *σ*_*x,y*_is the covariance of *x* and *y*, $C_{1}={\left(k_{1}L\right)}^{2}$ , $C_{1}={\left(k_{2}L\right)}^{2}$ , are constants to stabilize the division with a weak denominator; *L* is the dynamic range of the pixel-values. The results are summarized in Figure 4, which shows randomly sampled high-resolution projected and generated images. The SSIM values around the projection are notably low due to the projected images having a slight defocus, which renders SSIM particularly sensitive to fluctuations caused by defocus-induced phase reversals.

**FIGURE 5:**
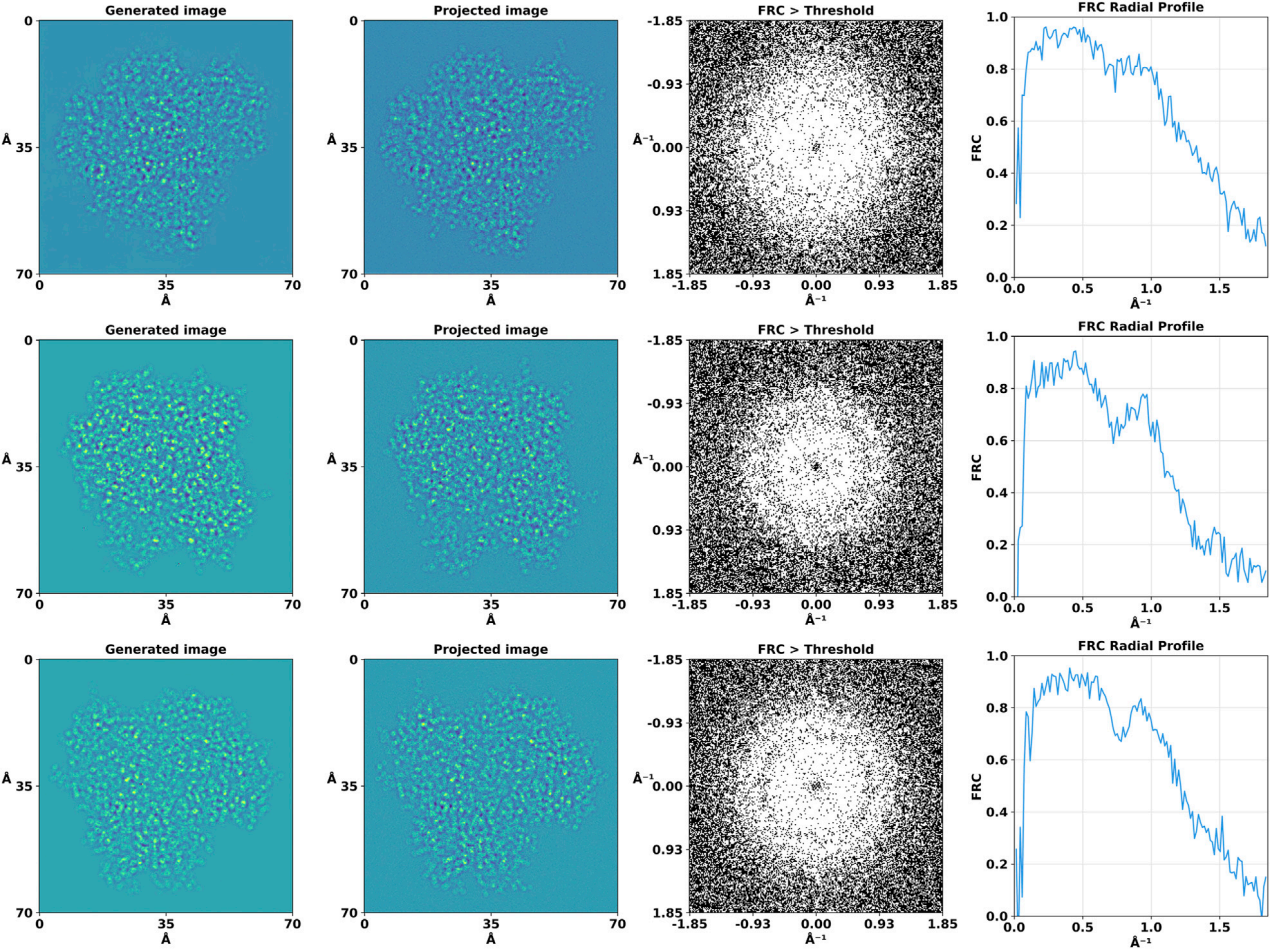
Fourier Ring Correlation (FRC) analysis of the high-resolution projection and generated images. For the purpose of this analysis, the binary mask threshold value for significant correlation was set at 0.5, allowing us to discern areas of high similarity between the compared images.

To quantitatively evaluate the degree of similarity between our generated images and their projected counterparts, we employed Fourier Ring Correlation (FRC) analysis (Koho et al., 2019). FRC provides a frequency domain metric for correlation at various scales between pairs of 2D images. Each image was Fourier transformed and the FRC was computed by normalizing the cross-spectrum of the two Fourier-transformed images by the geometric mean of their power spectra. A binary map was created to visualize the regions where the FRC values surpassed a 0.5 threshold (Figure 5).

**FIGURE 6:**
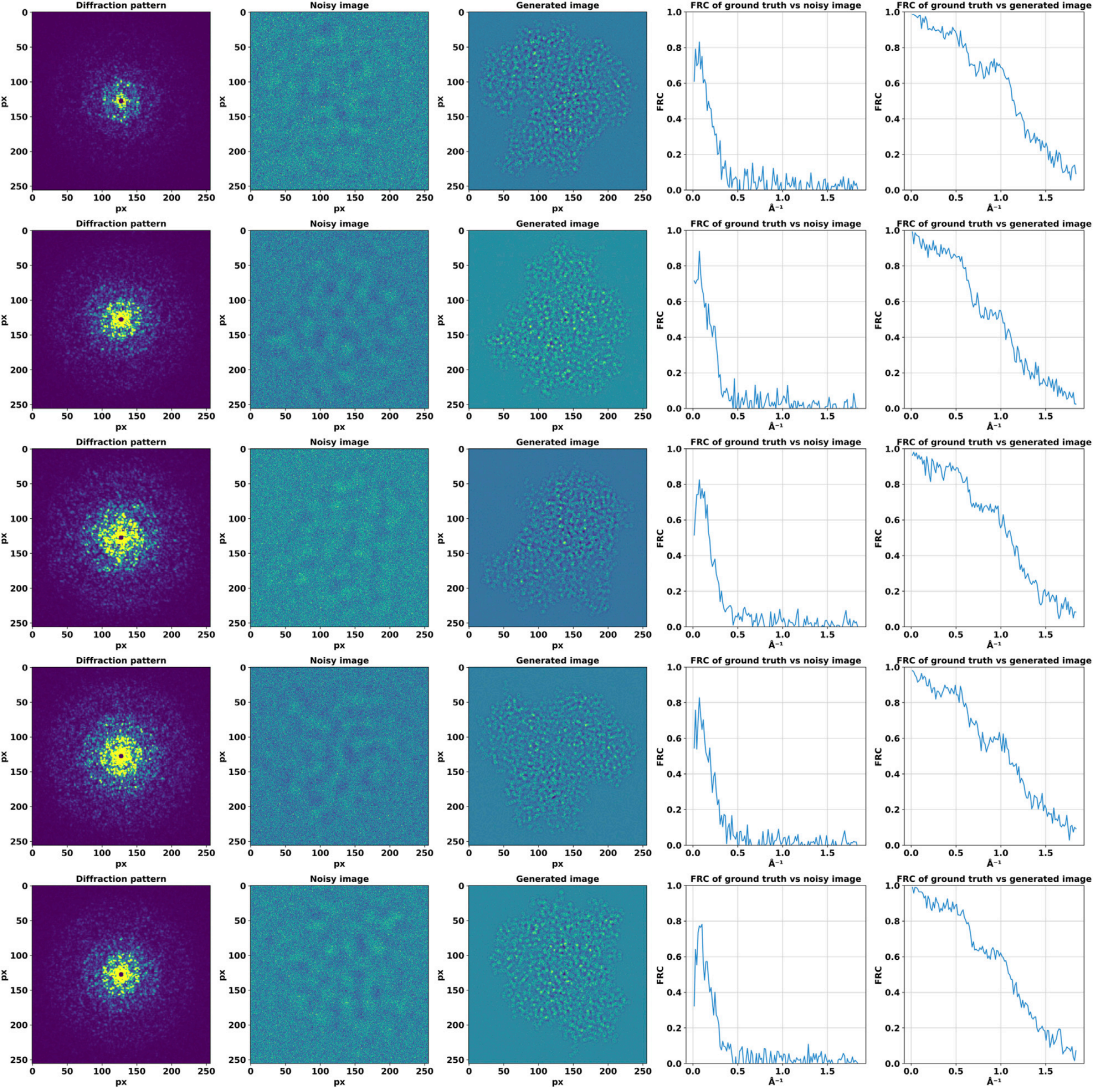
Fourier Ring Correlation (FRC) analysis of the high-resolution projections with noisy and generated images. First column: high-resolution diffraction patterns. Second column: low-resolution, defocused images. Third column: images generated by the generator using these inputs. Fourth column: FRC between ground truth (high-resolution projection) and noisy images. Fifth column: FRC between ground truth (high-resolution projection) and generated images.

In addition to the 2D analysis, we computed the 1D FRC curves by averaging the FRC values over concentric rings in the frequency domain. We also used FRC analysis to reveal the information gain provided by the DiffraGAN by comparing the high-resolution projections (ground truth) with noisy and generated images (Figure 6).

The FRC analysis revealed a high degree of correlation at lower and medium spatial frequencies, as evidenced by the central region where the FRC exceeded this threshold. This observation suggests that the generated and projected images share significant structural features to a high resolution of approximately 1Å. The correlation diminished slowly at higher spatial frequencies which could be also caused by defocus induced phase reversals. The 1D FRC profiles confirmed the trends observed in the 2D analysis, with a drop in correlation coefficients beyond a certain spatial frequency, while still being high at 1Å, thereby quantitatively delineating the resolution limits of our generated images relative to their projected counterparts.

**FIGURE 7:**
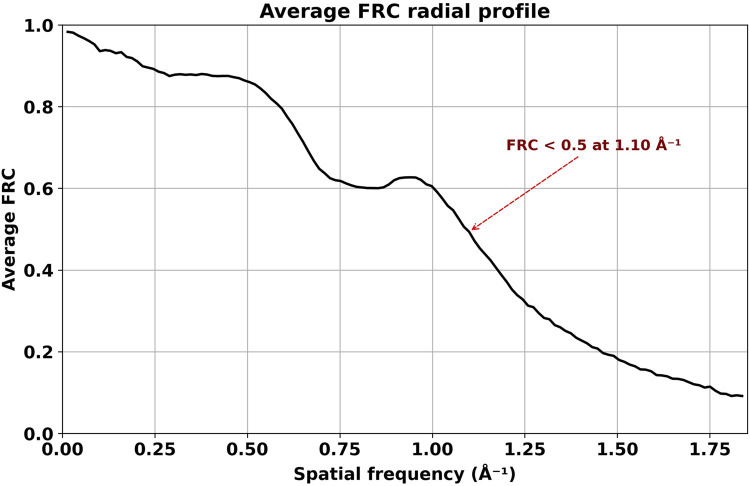
Average FRC calculated from DiffraGAN-generated model images and ground truth high-resolution projection images of the first test protein (PDB ID: 1AUP). The FRC between model and ground truth does not reach zero at the highest resolution depicted in the graph, indicating that DiffraGAN managed to extract some phase information close to the Nyquist frequency.

The average FRC, calculated using all 1078 angular orientations, drops below 0.5 at 0.90 Å (Figure 7). The combined visualizations and statistical analyses presented in Figures 4–6 confirm the quality and similarity of generated images in comparison to high-resolution projections.

**FIGURE 8:**
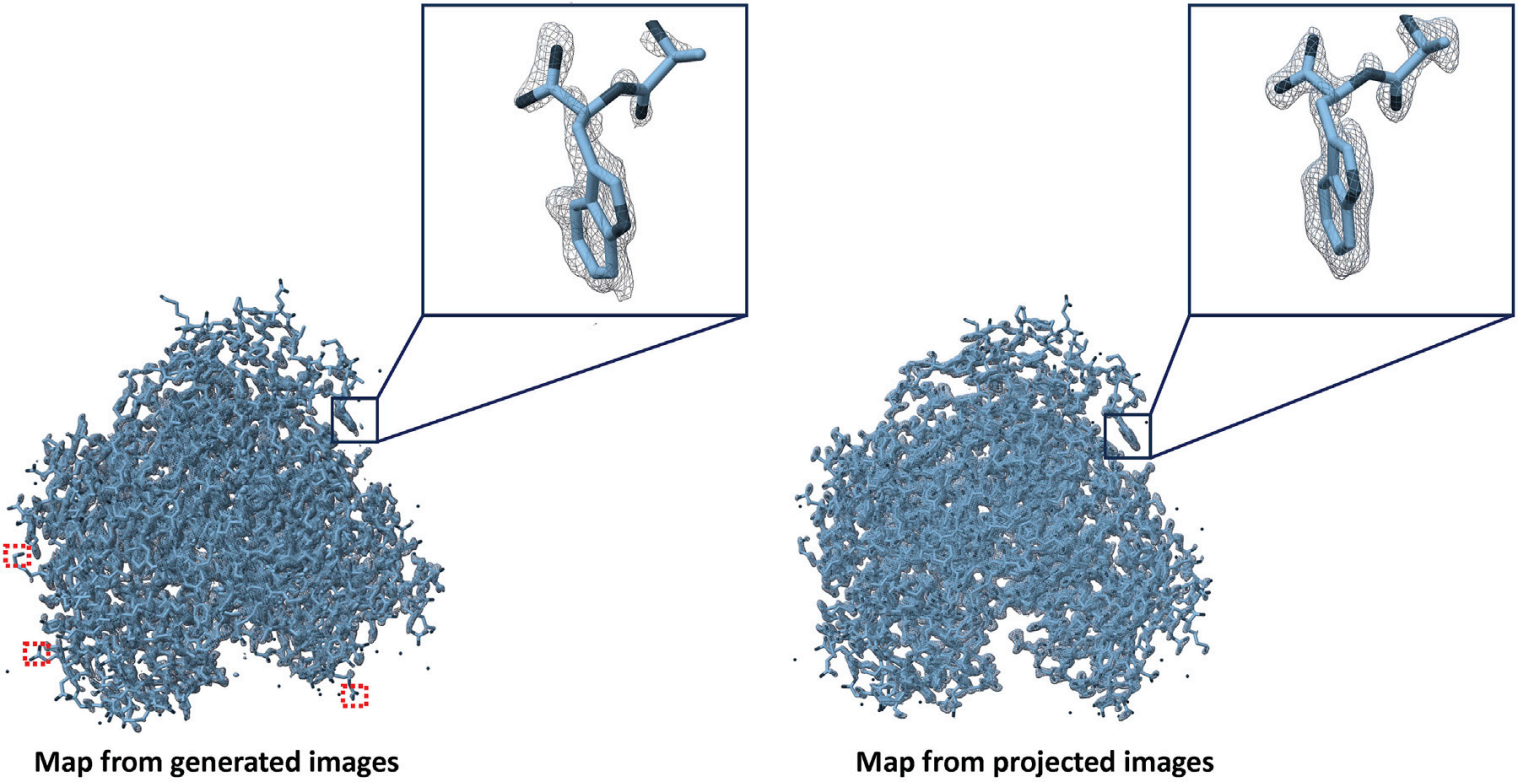
Maps created from generated and high-resolution projection images of the first test protein (PDB ID: 1AUP). Red squares represent absence of map density in case of generated images if present in case of projected ones.

8,000 high-resolution projection and DiffraGAN-generated images were then used for two 3D reconstructions of the simulated protein with RELION-4.0 (Kimanius et al., 2021). We reconstructed each set of high-resolution images *ab initio*. Both sets of images gave rise to a directly interpretable initial model and were refined to convergence using gold-standard refinement procedure without modification. As the generated images are not subject to CTF-related aberrations, we turned CTF correction off in *ab initio* model generation and in refinement. We fitted the PDB model in the maps using ChimeraX (Goddard et al., 2018) as shown in Figure 8. DiffraGAN sometimes struggles to generate clear outer shape features and side chain distributions, which can translate into poor map/model fit on the periphery of the map, highlighted in square red dots. DiffraGAN occasionally introduces “hot” pixels in regions beyond the protein’s structure, thereby possibly complicating the 3D refinement process. Nevertheless, the workflow creates a highly interpretable map that can be used for model fitting.

## 4 Discussion

GANs are a powerful class of deep learning models that have been applied to a wide range of tasks, including image generation and natural language processing. Recently, GANs have also been used for protein structure prediction (Strokach and Kim, 2022; Ingraham et al., 2023).

In this paper, we explore a new approach that could make phasing of coherent diffraction patterns of non-crystalline specimens much simpler, and possibly the same is true for more complex samples.

One key advantage of using conditional GANs for protein structure prediction from diffraction data and low-resolution features is their ability to capture the underlying feature distribution, which allows the model to generate diverse and realistic protein structures rather than just predicting a single most likely structure. This can be particularly useful for predicting the structures of proteins with multiple possible conformations, such as those involved in protein-protein interactions.

It appears that DiffraGAN can generate accurate images from corresponding single molecule diffraction patterns and low-resolution features of previously unseen proteins. All that is required, is a single molecule’s diffraction pattern, its low-resolution image, and statistics concerning the general distribution of projected protein density. In our case, once DiffraGAN converged, it could map diffraction patterns and defocused images to the corresponding high-resolution image with reasonable accuracy. The model was reliably producing similar images for the same, slightly augmented input and corresponding to the high-resolution projection image, showcasing its stability and robustness in generating data (Supplementary Figure S4). While there is theoretically no limit to the number of proteins the underlying GAN structure can be trained on, the performance of the GAN may decrease as the number of proteins that it is trained on increases. However, this will increase the generalization capacity of the network, as increasing the heterogeneity of the training data decreases the chances for overfitting. While theoretically such a function could be created, the increase in computational requirements could outweigh the potential benefits. It may be more beneficial to train multiple specialised GANs. This is equivalent to giving the GAN some additional information, like the molecular weight, or whether it concerns a membrane protein. How advantageous this approach could be has yet to be explored, however, from the test results (Supplementary Figures S1, S2), it is clear that the trained GAN better maps the diffraction patterns from the proteins whose shape and size resembles that of most of the proteins it was trained on, while performing worse when trying to map the diffraction patterns from the protein whose shape is the furthest away from the rest.

DiffraGAN has certain limitations. Firstly, the protein models are simulated in a vacuum and the calculated diffraction data did not simulate Poisson noise due to counting statistics, so appropriate denoising should be ensured before employing this method. We chose defocus to be relatively low, because we had to use relatively small proteins to be able to do the simulation and training in a reasonable time frame. DiffraGAN been exclusively trained on simulated asymmetric proteins up to 40 kDa in size and with a single defocus value. When tested with different defocus values, DiffraGAN can still reliably generate images from 1,500 Å defocused images (Supplementary Figures S5, S6); however, the performance drops significantly when the defocus is increased to 2,000 Å (Supplementary Figure S7). We anticipate that for larger protein complexes, higher defocus levels will produce similar results and providing the model with a training dataset that includes different defocus values could increase the model’s reliability range. We are currently conducting tests to verify this assumption. We also anticipate that our current methodology, which has been optimized for smaller proteins, might require substantial modification to accommodate the unique requirements of membrane proteins. In addition, when DiffraGAN is applied to smaller proteins below 10 kDa, as detailed in the Supplementary Figure S3, and generated using the exact same procedure, there are more discrepancies between DiffraGAN results and the actual projections. This is because the same level of noise tends to obscure more details in smaller proteins compared to larger ones and uniform data generation and rescaling process disproportionately impacts the diffraction patterns of smaller proteins, often leading to a greater loss of resolution and detail. By using diffraction data collection in conjunction with cGAN image generation, structural analysis of proteins with electron microscopy can be extended to proteins that were previously infeasible to study using cryo-EM due to lack of SNR owing to their small size.

Our results suggest that phase extension from low resolution images to high resolution is feasible with electron diffraction data. Unlike traditional cryo-EM, which focuses on high-resolution image details, our goal is to capture those details in the diffraction patterns. We are developing a general package for single molecule electron diffraction (simED), in which first a sample is scanned with a narrow beam, and high-resolution diffraction data are collected on the fly. Then an overview of the sample is collected as an image, using a high dose and defocus, aiming for localization and low-resolution contours of any particles and phasing using DiffraGAN (Supplementary Material S2). The additional information that allows this extension will in part be provided by the same restraints and constraints that allow phase extension in protein crystallography, like histogram matching, solvent flatness combined with the molecular contours and atomicity at high resolution. We speculate that DiffraGAN has discovered additional restraints in the data that are associated with macromolecular structures and may include elements of secondary structure and other complicated statistical correlations that are present in the data.

We are currently testing our methods on experimental data and are investigating to what extent it is limited by additional sources of experimental noise. Our *in silico* results are promising, indicating that the use of GANs for phasing may have the power to revolutionise methods in protein detection and structure elucidation by offering a potential solution to the phase problem.

## Data Availability

The data and code presented in the study are deposited in the GitHub repository, accession link: https://github.com/senikm/diffraGAN.

## Footnotes

1
https://github.com/openmm/pdbfixer
