# Supplementary material for: DiffraGAN: a conditional generative adversarial network for phasing single molecule diffraction data to atomic resolution
Source: Front Mol Biosci. 2024 May 22;11:1386963. doi: 10.3389/fmolb.2024.1386963 (PMC11150865; doi:10.3389/fmolb.2024.1386963)
Supplement: Supplementary file 1 [file DataSheet2.pdf]

Supplementary Material 2

DiffraGAN: a conditional generative adversarial network for phasing single molecule diffraction data to atomic resolution

S. Matinyan[1], P. Filipcik[1], E. van Genderen[2], J.P. Abrahams[1,2*]

1. Biozentrum, Basel University, Basel, Switzerland
2. Paul Scherrer Institute, Villigen, Switzerland

*** Correspondence:**
Corresponding Author
jp.abrahams@unibas.ch

1. Data collection

We used a JEOL F200 TEM with a Schottky FEG, operated at 200 keV, fitted with a CEOS CEFID energy filter and an ASI Cheetah M3 retractable hybrid pixel detector. First, standard alignment has been executed with the goal to achieve the minimum diameter parallel beam possible. We subsequently configured different sets of Intermediate Lens (IL) values, enabling us to make the detector conjugate with either the image plane, or the back focal plane. In simED approach, we initially collect a scan of diffraction patterns from partially overlapping probe positions (Figure S2). Then, after setting up imaging configuration, we proceed to capture images from the same scan area. We performed beam diameter and pixel size calibration prior to data collection. JEOL F200 TEM has an effective scan - descan system that compensates for tilt-shift variations above the sample plane using a second set of deflectors located beneath the sample. To adapt the system for narrow beam electron diffraction, we refined the descan process to minimize beam drift on the detector during scanning.

2. Data analysis use case using gold nanoparticles

2.1. Polycrystalline gold

For our initial tests, we used a polycrystalline gold (Au) sample, known for its specific lattice spacing, placed on a thin carbon replica with periodicity of 463 nm. We randomly selected a scan area within our field of view (Figure S1 a). We used virtual annular detector to reveal scan area via summation over concentric rings (Figure S1 b).

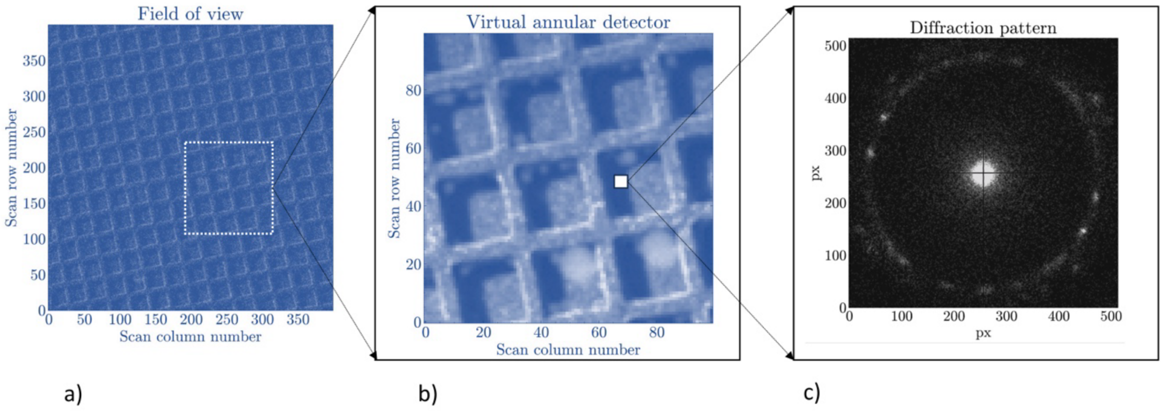

Figure S1. Analysis of narrow beam diffraction patterns from polycrystalline sample. a) Field of scan area b) Virtual annular detector has been used to allow summation over concentric rings. c) Individual diffraction pattern.

2.2. Image stitching

To evaluate the field of view and facilitate phase retrieval as suggested by DiffraGAN from the second scan, we developed image stitching across various scan positions. After finding the beam center, the image positions were masked with n-pixel radius and a square crop is applied within this circle (Figure S2 b). The resulting square images are overlaid to identify the optimal offset (Figure S2 c).

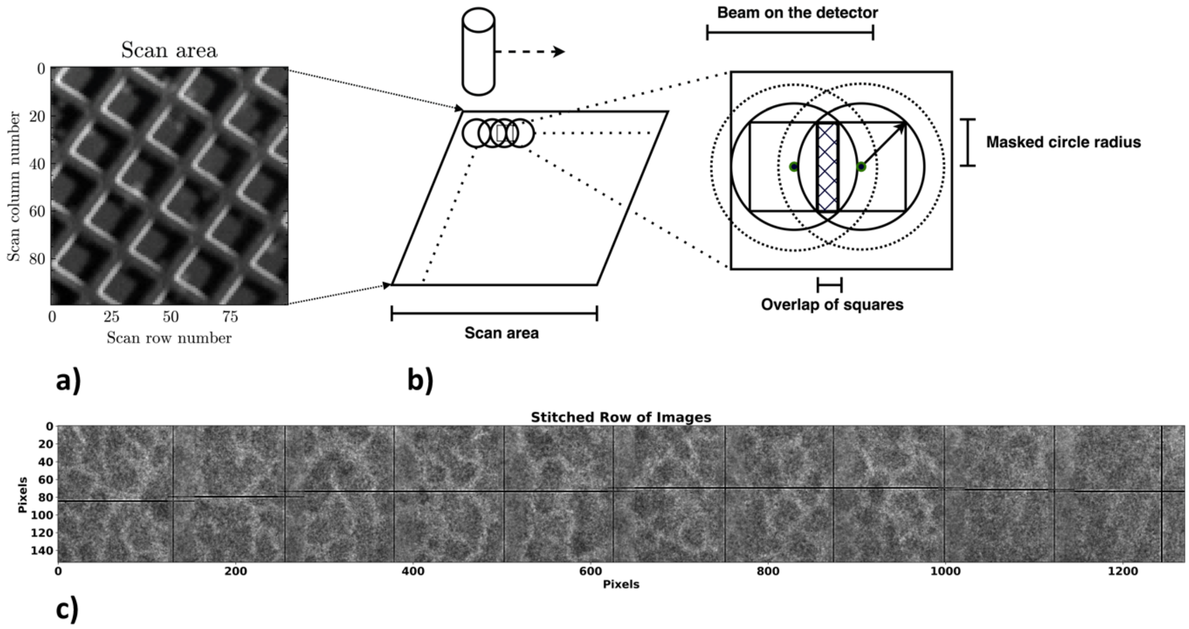

Figure S2. Stitching process a) Generated scan area using virtual annular detector. b) Stitching scheme showing circular and square crops and overlap region. c) Outcome of the stitching algorithm.

3. Machine learning based identification of positions

We previously published a methodology to select amorphous ice positions from carbon and crystalline ice (Figure S3 c) (Matinyan et al., 2023), thus reducing the amount of data needed to be analyzed. Currently, the approach uses ResNet-34 architecture as the main model (Szegedy et al., 2016) We created a Graphical User Interface (GUI) (Figure S3 b), which allows us effectively label the positions of the main classes from the virtual DF images. The model has the capacity to be retrained and fine-tuned upon change of experimental setup and when there is a data drift.

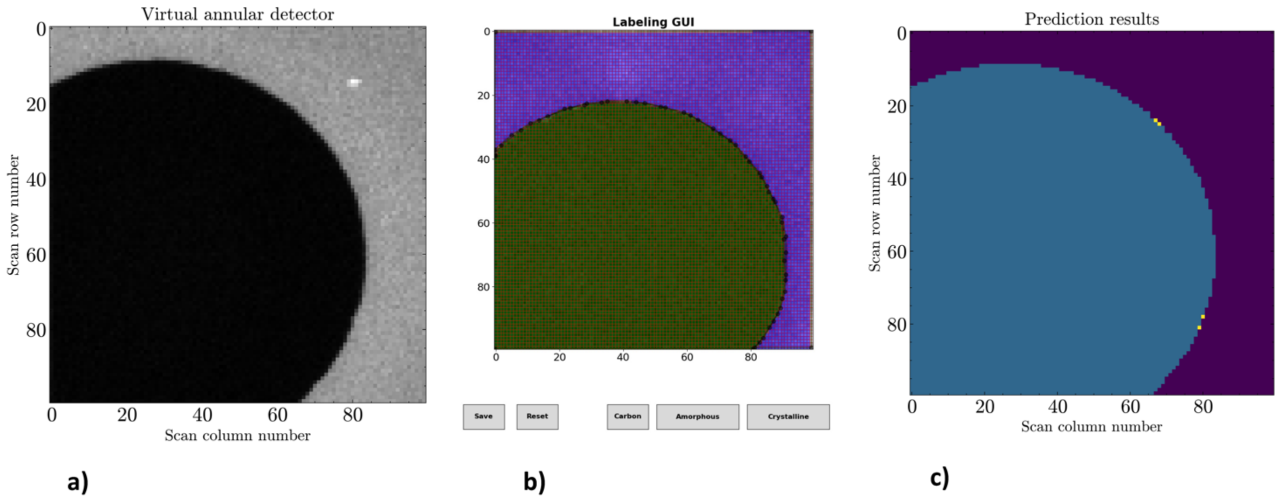

Figure S3. a) Virtual annular detector to reveal the scan map. b) Custom GUI to efficiently label the scan points. c) Prediction results of ResNet-34 architecture.

We are currently recording data from protein samples with subsequent scan position selection and image stitching as described above. Once the experimental setup is steady enough, we will proceed to phasing of individual diffraction frames using DiffraGAN as one of the main approaches.

3. References

Matinyan, S., Demir, B., Filipcik, P., Abrahams, J. P., and Van Genderen, E. (2023). Machine learning for classifying narrow-beam electron diffraction data. *Acta Crystallogr. Sect. A Found. Adv.* 79, 360–368. doi: 10.1107/S2053273323004680/

Szegedy, C., Ioffe, S., Vanhoucke, V., and Alemi, A. (2016). Inception-v4, Inception-ResNet and the Impact of Residual Connections on Learning. *31st AAAI Conf. Artif. Intell. AAAI 2017*, 4278–4284. doi: 10.48550/arXiv.1602.07261
