# Supplementary material for: DiffraGAN: a conditional generative adversarial network for phasing single molecule diffraction data to atomic resolution
Source: Front Mol Biosci. 2024 May 22;11:1386963. doi: 10.3389/fmolb.2024.1386963 (PMC11150865; doi:10.3389/fmolb.2024.1386963)
Supplement: Supplementary file 2 [file DataSheet1.pdf]

Supplementary Material 1

DiffraGAN: a conditional generative adversarial network for phasing single molecule diffraction data to atomic resolution

S. Matinyan[1], P. Filipcik[1], E. van Genderen[2], J.P. Abrahams[1,2*]

1. Biozentrum, Basel University, Basel, Switzerland
2. Paul Scherrer Institute, Villigen, Switzerland

*** Correspondence:**
Corresponding Author
jp.abrahams@unibas.ch

1 DiffraGAN generated images for other test proteins

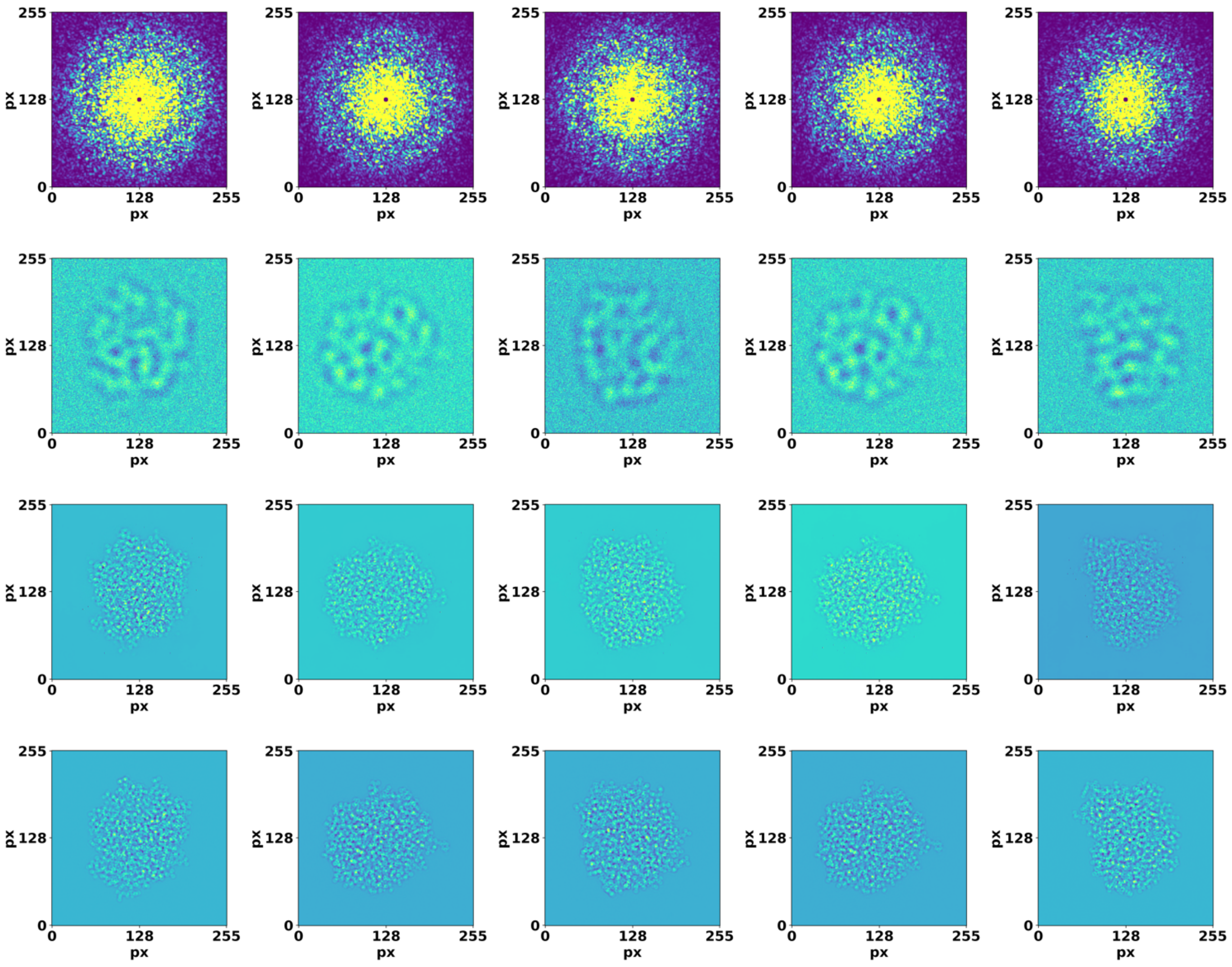

Figure S1. Validation of DiffraGAN using diffraction and image data from second test protein. Top row: input high resolution diffraction patterns. Second row: input low resolution, defocused images. Third row: images generated by the generator using these inputs. Bottom row: ground truth target images. The axes show pixel number.

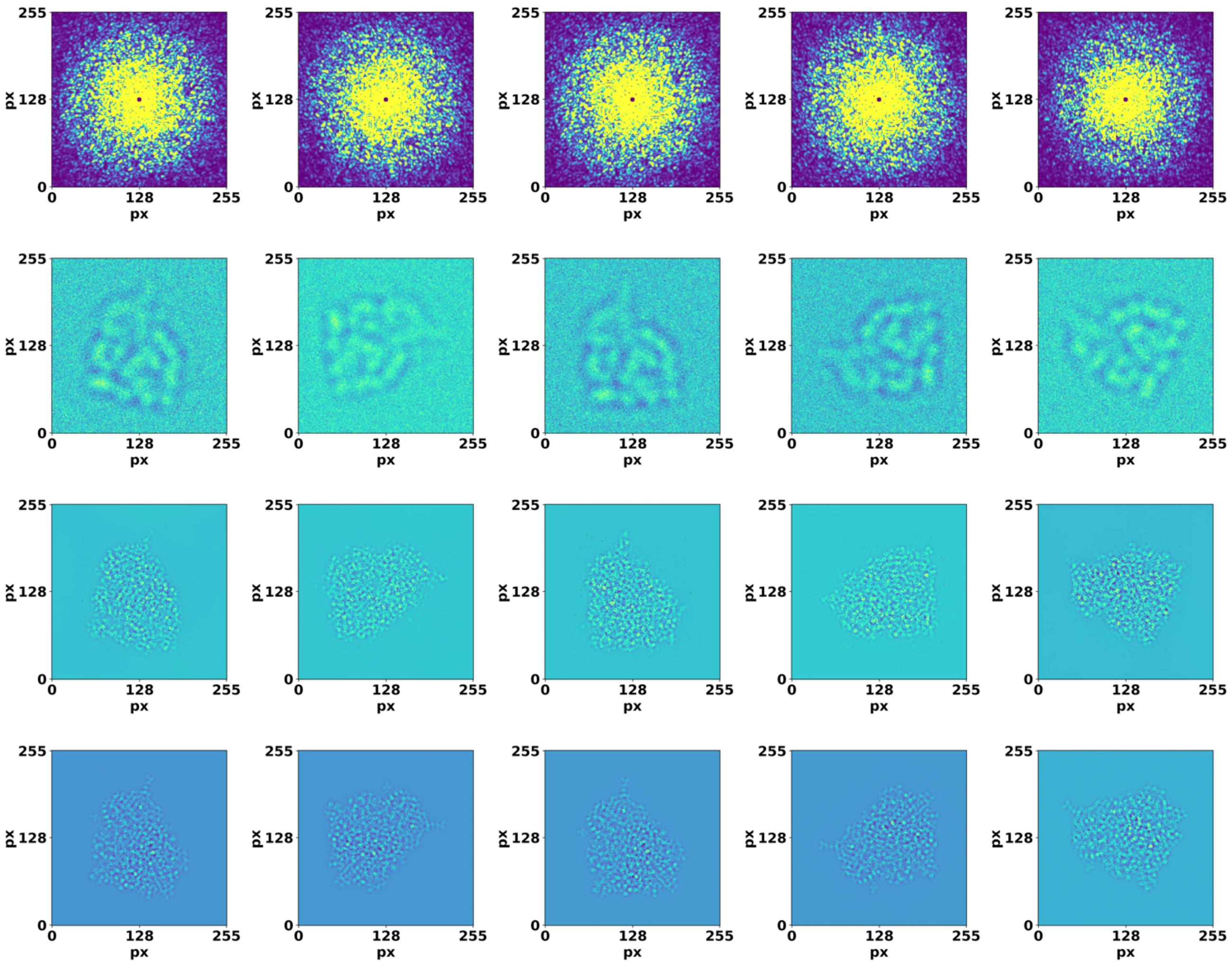

Figure S2. Validation of DiffraGAN using diffraction and image data from third test protein. Top row: input high resolution diffraction patterns. Second row: input low resolution, defocused images. Third row: images generated by the generator using these inputs. Bottom row: ground truth target images. The axes show pixel number.

2 DiffraGAN generated images for smaller proteins below 10kDa

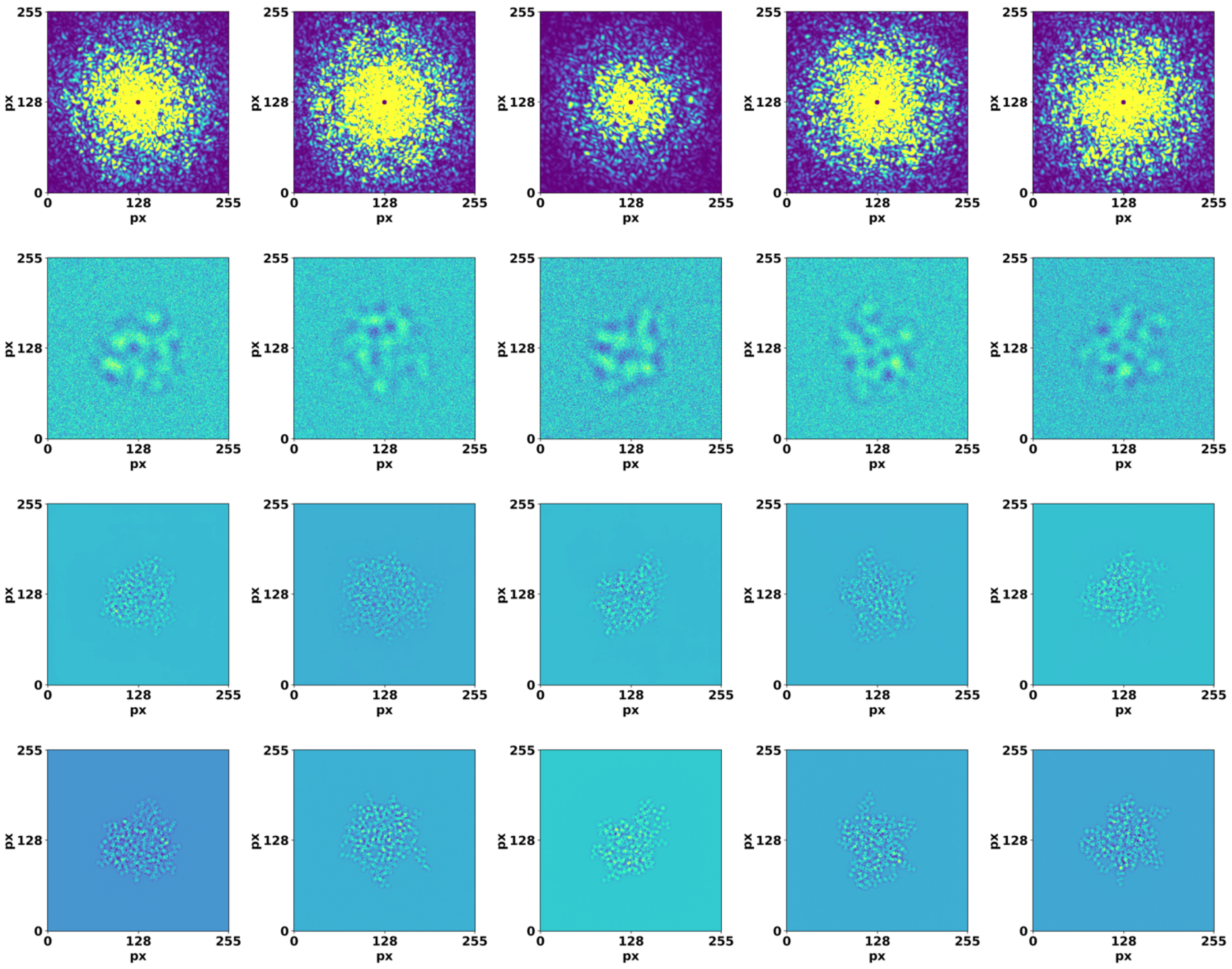

Figure S3. Validation of DiffraGAN using diffraction and image data from test protein below 10 kDa. Top row: input high resolution diffraction patterns. Second row: input low resolution, defocused images. Third row: images generated by the generator using these inputs. Bottom row: ground truth target images. The axes show pixel number.

3. DiffraGAN intra-class variability assessment

To assess the intra-class variability of our generative model, we performed a series of experiments where multiple outputs were generated from the same but slightly augmented set of inputs. The objective was to determine the degree of variation in the model's output when exposed to identical input conditions.

Randomly selected batch of samples from the test dataset were used to generate several outputs from each of these samples (Figure S4).

The intra-class variability has been assessed with SSIM metric, where in all pairs of intra-class images SSIM was higher than 0.98, indicating a high degree of similarity among the generated images (Figure S4).

These findings suggest that our generative model maintains a high level of consistency in its output.

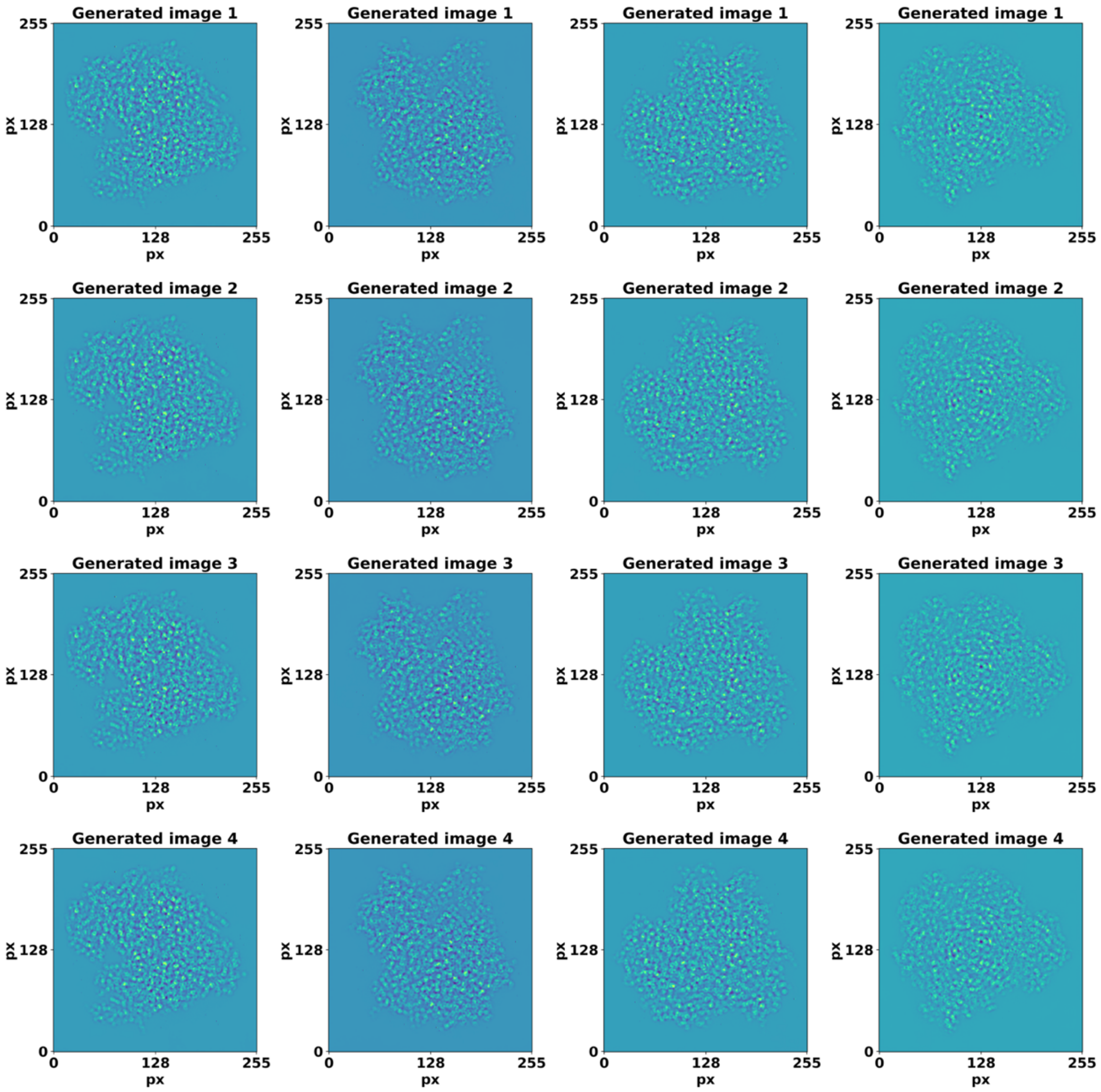

Figure S4. The plot visualizes the sets of generated images for each input sample in columns and illustrates subtle variations and the overall consistency in the model's output.

4 Ways to increase the capacity of generation process.

The following are ways by which the underlying structure of this GAN can be altered to increase its generator's accuracy:

1. *More Filters.*

   Adding more filters in the Conv2D layers in both or either of the discriminator and generator increases the number of parameters in the GAN that are available to train, and so the GAN

can perform more nuanced computations. The downside of adding more filters is that larger GANs take longer to train. In addition, adding more filters could lead to filter redundancy, though it is not yet clear where that point is in this case.

2. *Label Smoothing.*
   In a traditional GAN, the discriminator is trained to label 'real' images with ones and generated images with zeros. Changing the values of these labels (e.g. using 0.2 as the 'real' label and 0.8 as the generated label) changes the weighting of penalties on the generator and discriminator, and thus can change their training trajectories and the outcomes of training.

3. *Adding/ Removing Batch Normalisation and Dropout.*
   Adding batch normalisation and/ or dropout layers to the discriminator or removing some from the generator could alter the training trajectory and outcome. These alterations could end up worsening its performance. One example of this is adding a batch normalisation layer after every Conv2D layer in the discriminator. Regardless of the number of epochs of training, this caused the generator to produce non-stable outputs. This addition also delayed GAN's convergence. After adding a batch normalisation layer before each LeakyReLU layer in the generator, once the GAN converged, the generator produced more reasonable outputs.

4. *Changing The Size of The Discriminator Output.*
   As discussed in the method section, in traditional GANs, the output of the discriminator is 1x1 (one 'decision' per input image) while the discriminator structure used here has n-pixel patch structure. Increasing the size of the discriminator's output shape has been observed to reduce blurring, at the expense of increasing the frequency of 'hallucinated objects' appearing in images generated by the generator after training. While the discriminator is not guaranteed to behave this way when its output shape is varied, as before, the change could alter the training trajectory and outcome.

5. **DiffraGAN performance when given low resolution images with different defocus values**

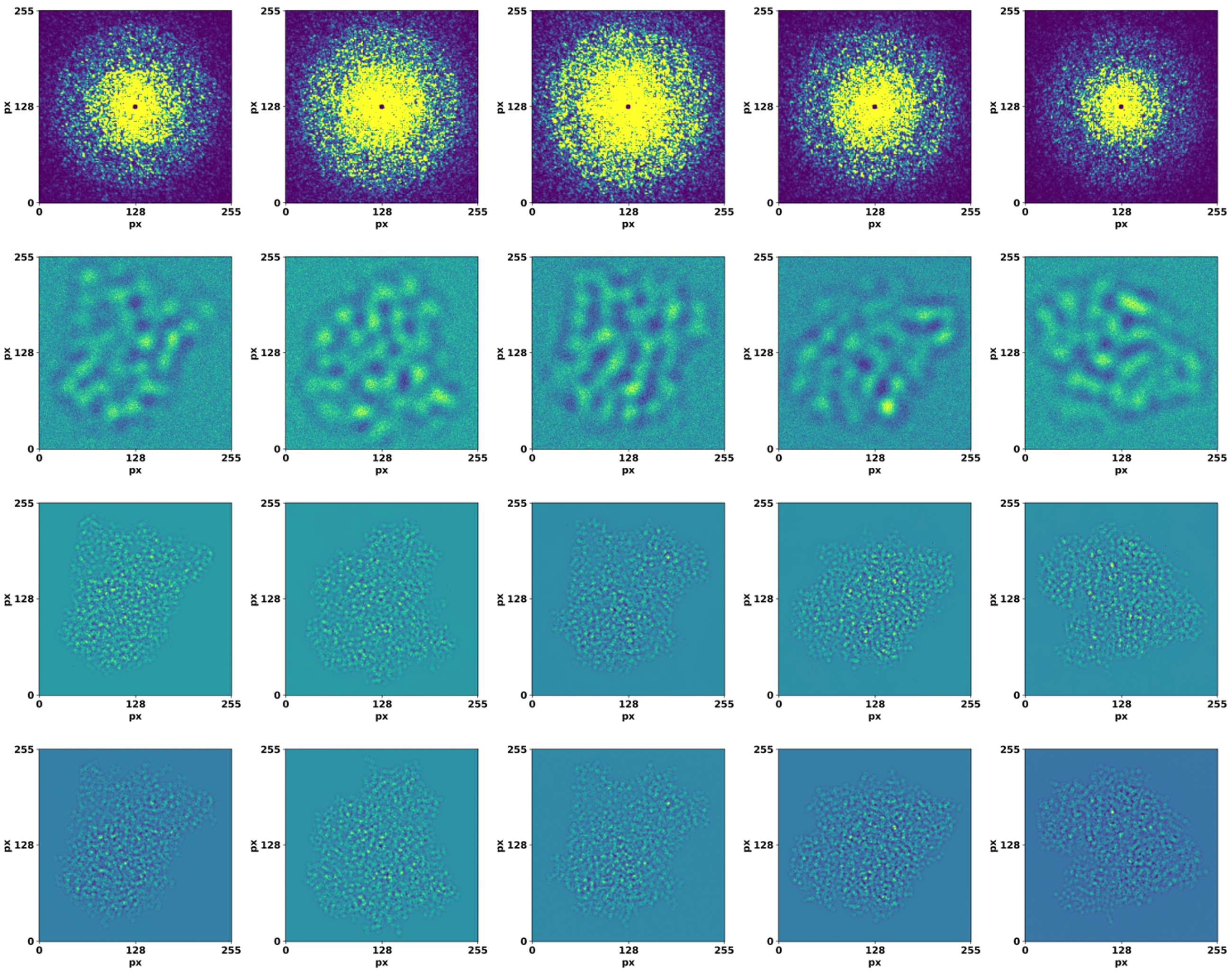

Figure S5. Validation of DiffraGAN using diffraction and image data from first test protein with a defocus value of 1500Å. Top row: input high resolution diffraction patterns. Second row: input low resolution, defocused images. Third row: images generated by the generator using these inputs. Bottom row: ground truth target images. The axes show pixel number.

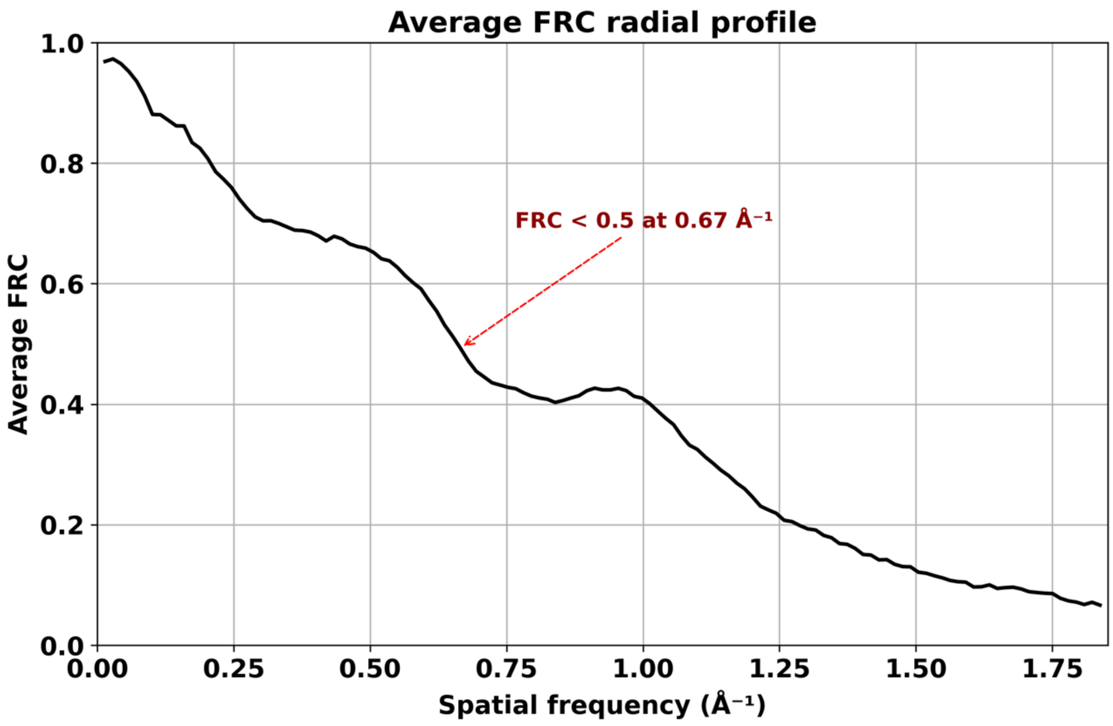

Figure S6. Average FRC calculated from DiffraGAN-generated images and ground truth high-resolution projection images of the first test protein (PDB ID: 1AUP). The low-resolution images have been generated with 1500 Å defocus.

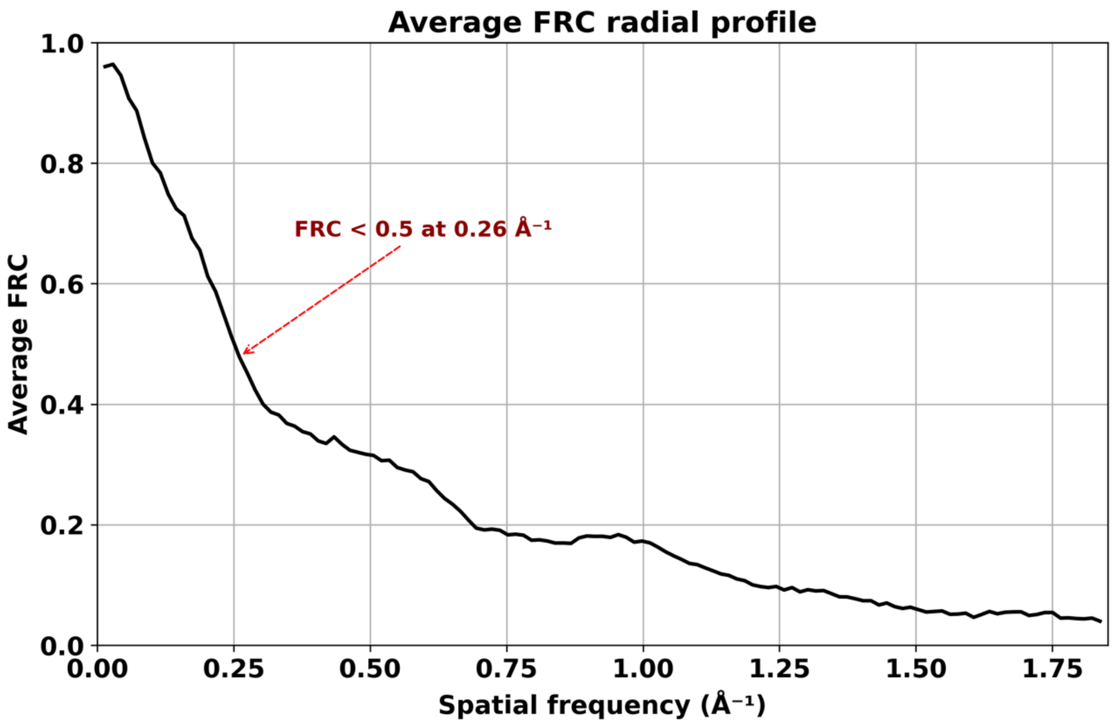

Figure S7. Average FRC calculated from DiffraGAN-generated images and ground truth high-resolution projection images of the first test protein (PDB ID: 1AUP). The low-resolution images have been generated with 2000 Å defocus.
